# Cassava Brown Streak Disease Response and Association With Agronomic Traits in Elite Nigerian Cassava Cultivars

**DOI:** 10.3389/fpls.2021.720532

**Published:** 2021-11-22

**Authors:** Chukwuka Ugochukwu Ano, Mildred Ochwo-Ssemakula, Angele Ibanda, Alfred Ozimati, Paul Gibson, Joseph Onyeka, Damian Njoku, Chiedozie Egesi, Robert S. Kawuki

**Affiliations:** ^1^College of Agricultural and Environmental Sciences, Makerere University, Kampala, Uganda; ^2^National Crops Resources Research Institute, Kampala, Uganda; ^3^National Root Crops Research Institute, Umudike, Nigeria; ^4^Makerere University Regional Center for Crop Improvement, Kampala, Uganda; ^5^International Institute of Tropical Agriculture, Ibadan, Nigeria; ^6^Cornell University Root Crops Research Institute, Ithaca, NY, United States

**Keywords:** breeding, cassava brown streak disease (CBSD), resistance screening, preemptive strategies, cassava germplasm, elite genotypes, cassava brown streak virus (CBSV), Ugandan cassava brown streak virus (UCBSV)

## Abstract

Cassava mosaic geminiviruses (CMGs) and cassava brown streak viruses (CBSVs) cause the highest yield losses in cassava production in Africa. In particular, cassava brown streak disease (CBSD) is and continues to be a significant constraint to optimal cassava production in Eastern and Southern Africa. While CBSD has not been reported in West Africa, its recent rapid spread and damage to cassava productivity in Eastern, and Southern Africa is alarming. The aim of this study was to evaluate Nigerian cassava genotypes in order to determine their responses to CBSD, in the event that it invades Nigeria, the world’s largest cassava producer. The study gathered information on whether useful CBSD resistance alleles are present in the elite Nigerian cassava accessions. A total of 1,980 full-sib cassava seedlings from 106 families were assessed in the field at the seedling stage for a year. A subset of 569 clones were selected and assessed for another year at the clonal stage in Namulonge, central Uganda, a known hotspot for CBSD screening. Results indicated that foliar and root incidences and severities varied significantly (*p* ≤ 0.01, *p* ≤ 0.001) except for CBSD foliar incidence at 6 months (CBSD_6*i*_). Highest and lowest plot-based heritability estimates for CBSD were registered for CBSD root severity (CBSD_*rs*_) (0.71) and CBSD_6*i*_ (0.5). Positive and highly significant correlations were noted between CBSD root incidence (CBSD_*ri*_) and CBSD_*rs*_ (*r* = 0.90^***^). Significant positive correlations were also noted between CBSD foliar severity at 3 months (CBSD_3*s*_) and CBSD foliar incidence at 6 months (CBSD_6*i*_) (*r* = 0.77^***^), CBSD_3*s*_ and CBSD_*rs*_ (*r* = 0.35^***^). Fresh root weight (Fresh_*RW*_) negatively correlated with CBSD_*ri*_ and CBSD_*rs*_, respectively (*r* = −0.21^***^ and *r* = −0.22^***^). Similarly, CBSD_3*s*_ correlated negatively with cassava mosaic disease severity at 3 (CMD_3*s*_) and 6 months (CMD_6*s*_), respectively (*r* = −0.25^***^ and *r* = −0.21^***^). Fifteen clones were selected using a non-weighted summation selection index for further screening. In conclusion, results revealed that the elite Nigerian accessions exhibited significant susceptibility to CBSD within 2 years of evaluation period. It is expected that this information will aid future breeding decisions for the improvement of CBSD resistance among the Nigerian cassava varieties.

## Introduction

Cassava (*Manihot esculenta* Crantz) is a perennial plant cultivated as an annual crop. It originated in Latin America and is widely grown across the subtropical and tropical regions of the world. It is an essential source of carbohydrate and a major staple food for over 800 million people globally (Hammond et al., 2013). Cassava is popular because of its hardy nature and ability to adapt to drought and low nutrient availability in the soil. The crop is also amenable to piecemeal harvesting for a period between 8 and 24 months after planting, an attribute that makes it popular among smallholder farmers. These inherent characteristics make cassava one of the most resilient food security crops of the 21st century. Approximately 70% of cassava is currently grown in Africa and Asia, with an estimated cultivated area of more than 22 million hectares (Food and Agricultural Organization of the United Nations [FAOSTAT], 2020). Africa accounts for 61% of 277 million tons of cassava production worldwide, most of which comes from Nigeria with an estimated 59 million tons (Food and Agricultural Organization of the United Nations [FAOSTAT], 2020).

Cassava is a basic staple food for more than 65% of the population in Nigeria who consume it in different forms at least once a day (Eke-okoro and Njoku, 2012). It is processed into over 50 food forms, such as garri, lafun, bread, flakes, and flour (Eke-okoro and Njoku, 2012). With the increasing demand for cassava for both food and non-food uses in Nigeria, efforts must be devoted to sustain and/or increase cassava production and productivity.

Significant cassava yield gaps are commonplace in most tropical countries. For example, in Nigeria, where more than 40 different varieties are grown by farmers (Bankole, 2019), the national average yield of 13.6 metric tons per hectare reflects a shortfall of 65.9% when compared with potential yield estimates of 40 metric tons per hectare (Food and Agricultural Organization of the United Nations [FAOSTAT], 2020). This yield gap is due to an array of biotic and abiotic constraints, notably poor agronomic practices, susceptibility to prevalent pests and diseases, drought, a deficit of clean planting materials, and drudgery associated with most farm operations. Among these constraints, diseases are by far the main impediments to optimal cassava production (Ntawuruhunga et al., 2013).

In Africa, cassava production is mostly limited by two viral diseases: cassava mosaic disease (CMD) and cassava brown streak disease (CBSD). CMD causes yield losses of up to 70% in susceptible varieties and can kill or stunt plants to varying degrees (Tomlinson et al., 2018). CBSD causes yield losses of up to 100% in susceptible varieties and destroys the edible roots even when the rest of the plant looks healthy (Hillocks et al., 2008). CMD is distributed across the major African cassava growing belt and is considered one of the most severe and widespread diseases of cassava in Nigeria. In contrast, CBSD is restricted to Eastern, Central, and some parts of Southern Africa and is currently considered the world’s most dangerous threat to cassava production (Legg et al., 2011; Patil et al., 2014; Eni et al., 2018; Tomlinson et al., 2018).

Based on complete genome sequences, CBSD is caused by two distinct virus species: cassava brown streak virus (CBSV) and Ugandan cassava brown streak virus (UCBSV; Mbanzibwa et al., 2009; Winter et al., 2010). Both species belong to the genus *Ipomovirus* and family Potyviridae (Alicai et al., 2016) and can be found in low- and mid-altitude areas (Munga, 2008; Mrema, 2016). Symptomatically, CBSD constricts and necrotizes cassava roots, rendering them unpalatable and unmarketable. On most instances, blotchy yellow chlorosis or feathery chlorosis appears on the minor veins of the leaves of infected plants. Brown, round, or elongated streak-like lesions can also occur on the young green portion of the infected stems. These lesions develop in severe infections to cause dieback and possibly kill the whole plant. The most prevalent viruses that are causal agents of CMD in Africa are single-stranded DNA bipartite cassava mosaic begomoviruses (CMBs), African cassava mosaic virus (ACMV), and East African cassava mosaic virus (EACMV). In Nigeria, CMD reached epidemic status in the 1950s and breeding for its resistance began in 1970 (Hahn et al., 1980). Since then, CMD resistance has been a backbone for all cassava varieties being developed and released by the national breeding program.

Both CBSD and CMD are transmitted over short distances by the whitefly vector, *Bemisia tabaci* (Gennadius) (Maruthi et al., 2005), whereas they are transmitted over long distances through the transport of infected planting materials (Patil et al., 2015). Management practices for both diseases include planting of clean (symptomless) cassava cuttings, uprooting and destroying cassava plants showing disease symptoms, sterilization of farm implements, especially when cutting cassava stems for multiplication, and using tolerant/resistant varieties. Cultivation of tolerant/resistant varieties remains the most viable management practice (Munga, 2008).

Previously regarded as a low-altitude disease, CBSD has recently been confirmed to exist in both lowland and highland areas, with the recent reports indicating a wide spread of CBSD in Uganda, Kenya, Malawi, Burundi, Rwanda, and some parts of the Democratic Republic of Congo (DRC; Hillocks et al., 2002; Bigirimana et al., 2011; Hillocks and Maruthi, 2015; Chipeta et al., 2016; Koima et al., 2018; Munganyinka et al., 2018). With the existence of CBSD in DRC (Mulimbi et al., 2012; Casinga et al., 2021), there is a possibility of the disease spreading towards West Africa. The potential reach of CBSD into West Africa, a region with no previous reports of the disease, is considered disturbing.

Unlike the East African cassava breeding programs (Munga, 2008; Abaca et al., 2012; Kawuki et al., 2016; Ferguson et al., 2017), West African cassava germplasm has not been undergoing selection for CBSD resistance because of the absence of the disease in the region and is, thus, likely to exhibit susceptibility. A westward drift of CBSD (Bigirimana et al., 2011; Mulimbi et al., 2012; Casinga et al., 2021), as well as projected studies indicating the presence of CBSD in West Africa by 2030 (Jarvis et al., 2012), is a cause for great concern. The recent surge in *B. tabaci* populations in Eastern and Central Africa, which might have played a role in CBSD outbreaks in mid-altitude regions (Legg et al., 2011, 2014), equally troubling. Preemptive breeding strategies to avert future impact of CBSD in West Africa is, therefore, critical since cassava is a major staple and industry crop in the region. Thus, the objectives of this study were to (i) determine the reaction of elite Nigerian cassava genotypes to CBSD in Uganda and (ii) determine the relationship between CBSD field response and other important agronomic attributes in the Nigerian cassava genotypes assessed in Uganda.

## Materials and Methods

### Study Site

This study was conducted at the National Crops Resources Research Institute (NaCRRI), Namulonge (0.5232°N, 32.6158°E) which is located at an altitude of 1,200 m above sea level and characterized by the bimodal rainfall distribution and an average annual temperature ranging between 24 and 30°C (Sebuwufu et al., 2015). The soil is characterized by acidic ferralsols, with pH of below 6.5–7.0 (Sebuwufu et al., 2015). NaCRRI is located 27 km north of Kampala, the capital city of Uganda, and has a tropical wet and mild dry climate with annual rainfall ranging between 1,000 and 1,450 mm with slight humid condition (average: 65%) (Sebuwufu et al., 2015). Namulonge, geographically located in central Uganda, is considered a hotspot for CBSD screening (Alicai et al., 2007; Sebuwufu et al., 2015). The site is also associated with high *B. tabaci* populations (Kawuki et al., 2016). These characteristics qualify NaCRRI as an optimal site for CBSD and CMD resistance screening (Abaca et al., 2012). In this study, we, therefore, assessed the response of introduced Nigerian germplasm to CBSD infestation at NaCRRI, a known hotspot in Uganda.

### Test Materials

In response to the CBSD spread across Africa, National Root Crops Research Institute (NRCRI), Umudike, Nigeria, took the initiative to screen some crosses of elite-by-elite cassava genotypes in Uganda, a hotspot for CBSD. A total of 5,000 botanical seeds were generated from various biparental crosses involving 48 elite progenitors. The progenitors were part of Cycle 1 of the NRCRI cyclic population and were selected based on their yielding ability and resistance to CMD. Accordingly, these seeds were then shipped and planted in a seedling nursery at Namulonge. The progenies were screened in Uganda in order to guide the NRCRI breeding program, Nigeria, in developing breeding strategies for CBSD resistance. A total of 1,980 botanical seeds successfully emerged into seedlings. These 1,980 seedlings represented 106 families (Supplementary Table 1).

### Seedling Evaluation Trial

In a field trial, the 1,980 seedlings were planted during the second rainfall of 2018 (September/October) for an evaluation period of one year and was harvested during the second rainfall of 2019 (September/October). The field trial was established in a completely randomized design, comprising six blocks, with each block consisting of 33 ranges. Each row comprised of 10 individuals (unique genotypes). Since replication of seedlings at a seedling trial is not possible (Barandica et al., 2016), we stratified the families across blocks such that sibs were planted in at least three separate blocks. Seedlings were planted at a spacing of 1 m × 1 m. The clone TME 204, which is highly susceptible to CBSD, was planted along the experimental borders as spreaders to augment CBSD pressure in the evaluation plots. The experiment was carried out under rainfed conditions, without the application of pesticides and fertilizers, and was kept weed-free by regular hand weeding.

### Clonal Evaluation Trial

The clonal evaluation trial (CET) was established at NaCRRI, Namulonge, during the second rainfall of 2019 (September/October) for a one year evaluation period and was harvested during the second rainfall of 2020 (September/October). The clonal trial constituted 569 clones advanced from the seedling stage. Advancement from seedling evaluation trial (SET) to CET was based mainly on the ability of a clone to produce ample planting materials, i.e., at least 10 stem cuttings. This is because 10 stem cuttings were required to constitute a row, which would represent a single clone. Briefly, the trial was established in an augmented design, comprising 24 blocks; each clone was planted in a single row of 10 stem cuttings, at a spacing of 1 m × 1 m within rows. Within each block, the selected test clones were planted along with three randomized checks, namely, TME 204, NAROCASS 1, and Mkumba. TME 204 was the most susceptible check to CBSD, whereas NAROCASS 1 and Mkumba were tolerant checks, officially released in Uganda and Tanzania, respectively. TME 204 also acted as a spreader to augment CBSD pressure in the field trial. Similar to the seedling trial, the experiment was carried out under rainfed conditions, without the application of pesticides and fertilizers, and was kept weed-free by regular hand weeding.

### Data Collection

**FIGURE 1:**
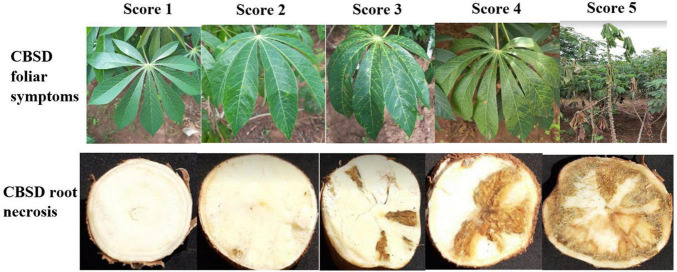
CBSD symptom scoring scale (1–5).

For both SET and CET trials, data were collected on CBSD and CMD. Foliar disease symptoms were assessed on each plant at three and six months after planting (MAP). For assessment of CBSD, plants were assigned disease severity scores based on the standard five-point scoring scale (Gondwe et al., 2003), where 1 = no apparent symptoms; 2 = slight foliar feathery chlorosis and no stem lesions; 3 = prominent foliar feathery chlorosis, mild-stem lesions, and no dieback; 4 = severe foliar feathery chlorosis, severe stem lesions, and no dieback; and 5 = defoliation, severe stem lesions, and dieback (Figure 1).

Similarly, CMD was assessed using the 1–5 scoring scale, where 1 = cassava plant showing no leaf symptom; 2 = mild distortion and mild chlorosis on the leaves; 3 = significant distortion and chlorosis on one-third of most leaves; 4 = extreme distortion and presence of mosaic patterns on two-third of most leaves and general reduction of leaf size; and 5 = very severe mosaic symptoms on all leaves, appearance of distortion, twisting, misshapen, and severe leaf reduction of most leaves accompanied by severe stunting of plants (Thresh and Cooter, 2005).

At 12MAP (during harvest), individual plants (in SET) and plots (in CET) were uprooted, and roots were sliced for CBSD root necrosis assessment. The CBSD root severity was assessed on all harvested roots, from a plant, using the 1–5 scoring scale (Gondwe et al., 2003), where 1 = no apparent necrosis, 2 ≤5% of root necrosis, 3 = 6–10% of root necrosis, 4 = 11–25% of root necrosis and mild root constrictions, and 5 ≥25% of root necrosis with severe root constriction (Figure 1).

Finally, for both SET and CET, data were collected on total carotenoid content and fresh root weight (FRW). The total carotenoid content was assessed visually using a qualitative 1–6 standard root color scoring scale developed by the International Center for Tropical Agriculture (CIAT), where 1 = white, 2 = light cream, 3 = cream, 4 = light yellow, 5 = yellow, and 6 = deep yellow. To estimate fresh root yield (FRYD) (tons ha^–1^) from FRW, we used the formula: FRYD = [(FRW/Number of plants harvested per plot) × 10,000]/1,000 (Tumuhimbise et al., 2014).

### Data Analyses

Datasets generated in the seedling trial were described using summary statistics in R (R Core Team, 2020). Relationships between CBSD severity and other evaluated key agronomic traits were visualized using the gg*_*scatter*_* function in R (R Core Team, 2020). The Pearson’s correlation test was conducted, and the coefficient of correlation was extracted using the *cor.test.* All the functions mentioned above are available in *ggpubr* package in R.

For clonal trial datasets, linear mixed model effects using *lme4* package in R was used to estimate variance components and, thus, to enable the estimation of best linear unbiased predictors (BLUPs). The BLUPs were computed to enable genotype comparison for the unbalanced dataset (Bernardo, 2010). The linear model used to generate the analysis of variance for single-site analysis was as follows:


$$Y_{ijk}=\mu +C_{i}+B_{j}+G_{k}+\varepsilon_{ijk},$$

where *Y*_*ijk*_ is the observed response of the *i*-th clone in the *j*-th block for the *k*-th test genotype, μ is the general mean of the genotypes, *C*_*i*_ is the fixed effect of the checks, *B*_*j*_ is the random effect of the block, *G*_*k*_ is the random effect of the test genotypes, and ε_*ijk*_ is the random error associated with the *ijk*-th observation. The broad-sense heritability estimates for each trait were calculated using the following formula:


$$H^{2}=\sigma^{2}G/\left(\sigma^{2}G+\sigma^{2}e\right),$$

where *H*^2^ is the broad-sense heritability, σ^2^*G* is the genotype variance, and σ^2^*e* is the error variance. To extract the variance components for computing broad-sense heritability, the effects of the block and test genotypes were considered random, whereas the check effects were considered fixed.

A non-weighted rank summation selection index (SI) was used to compare the performance of the test genotypes. Briefly, genotype BLUP values generated from CBSD foliar and root scores were ranked and subsequently summed up to compute the SI (Mulamba and Mock, 1978).

## Results

### Field Reaction of Cassava Genotypes at the Seedling Stage

**FIGURE 2:**
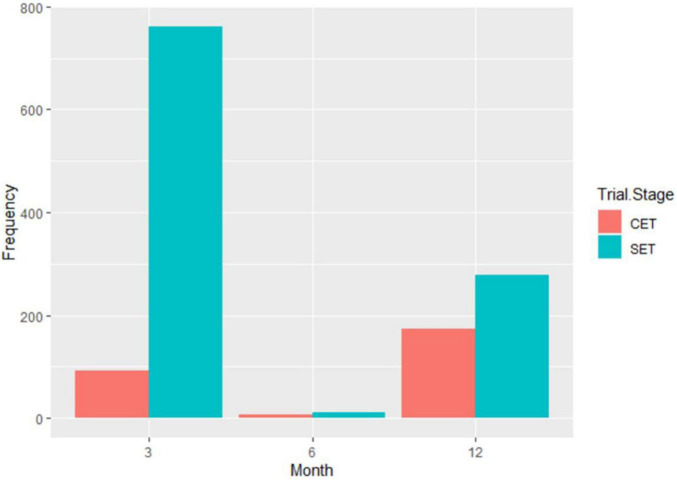
Dieback and death inflicted by CBSD on the Nigerian germplasm evaluated in Uganda at SET and CET.

**FIGURE 3:**
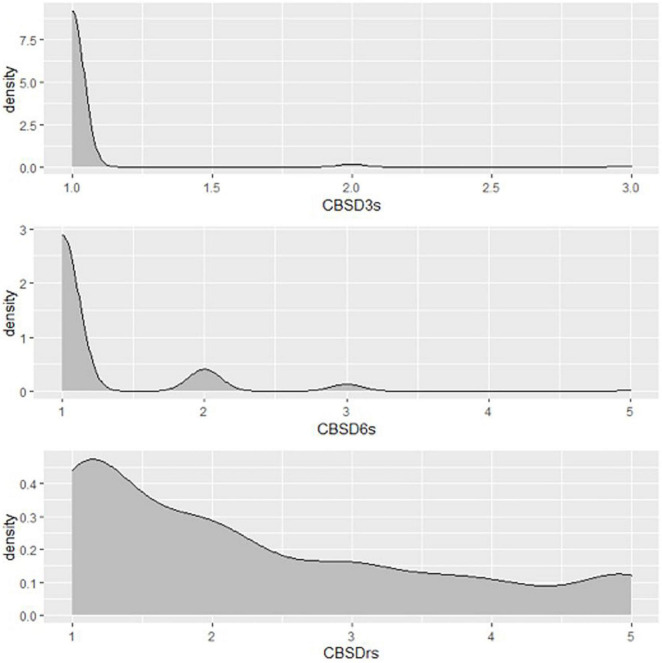
Distribution of CBSD foliar and root severity scores of Nigerian germplasm evaluated at SET in Namulonge, Uganda in 2018. CBSD_3*s*_, CBSD severity assessed at three months after planting, CBSD severity assessed at six months after planting, CBSD_*rs*_, CBSD root severity at 12MAP.

A total of 1,980 genotypes were planted and evaluated in the seedling trial. However, by the third month, 38% of the genotypes had succumbed to CBSD pressure (Figure 2). About 0.01% of genotypes were lost by the 6th month, and by the 12th month, an additional 15% of genotypes had succumbed to CBSD pressure. Hence, only 47% of the genotypes survived, and the root necrosis assessment were undertaken on them at 12MAP. For the genotypes that survived up to 3 months after planting (3MAP), the CBSD severity ranged from 1 to 3 at 3MAP, while at 6MAP and 12MAP, it ranged from 1 to 5 (Figure 3). Percentage CBSD root incidence ranged from 0 to 100%, with a mean score of 56.8. Mean severity scores for CBSD at 3MAP, 6MAP, and 12MAP were 1.03, 1.21, and 2.30, respectively.

**FIGURE 4:**
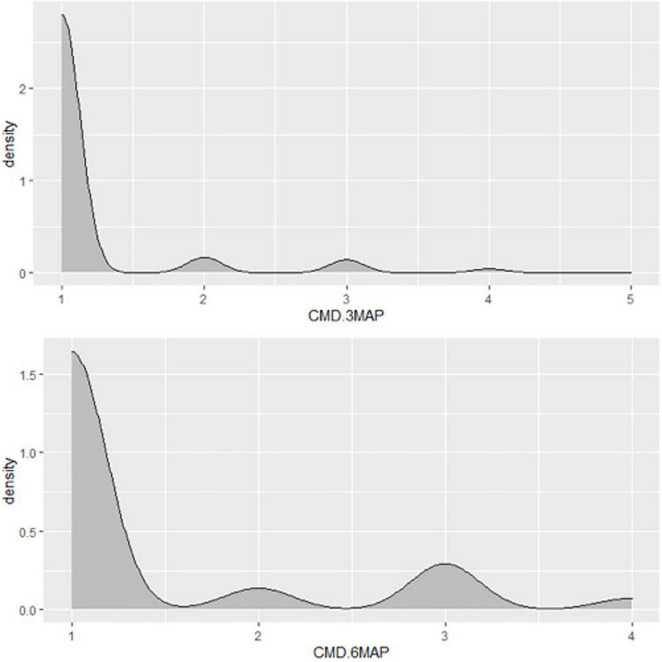
Distribution of CMD severity scores of Nigerian germplasm evaluated at SET in Namulonge, Uganda in 2018. CMD.3MAP, CMD severity assessed at three months after planting, CBMD severity assessed at six months after planting.

A total of 1,014 (out of 1,206) genotypes had low CBSD foliar severity score (≤1.5) at 6 months. In contrast, 330 out of 927 genotypes assessed during harvest exhibited low root necrosis severity score (≤1.5). Overall, 51% of the 927 accessions evaluated had yellow roots. Notably, there was a gradual increase in severity from 3MAP to 6MAP, with the highest average score at 12MAP (Supplementary Table 2).

### Response of Cassava Genotypes to Cassava Brown Streak Disease at the Clonal Stage

**FIGURE 5:**
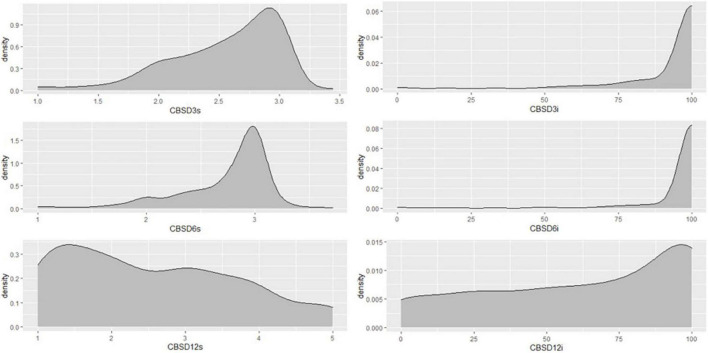
Distribution of CBSD foliar and root severities and incidences in Nigerian germplasm evaluated in clonal trial Uganda in 2019. CBSD_3*s*_, CBSD severity 3MAP; CBSD_6*s*_, CBSD severity 6MAP; CBSD_12s_, CBSD root severity at 12MAP; CBSD_3*i*_, CBSD incidence 3MAP; CBSD_6*i*_, CBSD incidence 6MAP; CBSD_12i_, CBSD root incidence at 12MAP. Analysis based on 297 genotypes.

**FIGURE 6:**
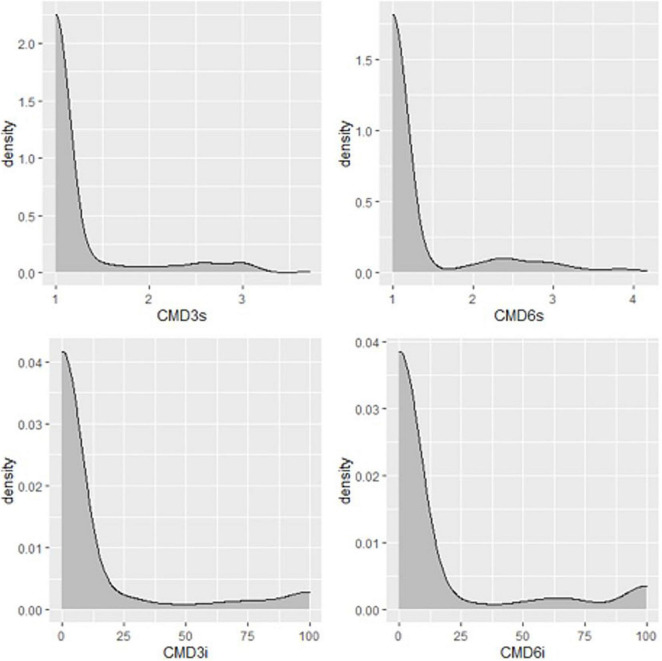
Distribution of CMD severities in Nigerian germplasm evaluated in clonal trial in Uganda in 2019. CMD_3*s*_, CMD severity assessed at three months after planting, CBMD severity assessed at six months after planting, CMD incidence assessed at three months after planting, CMD incidence assessed at six months after planting.

A total of 569 clones were selected from the seedling trial for further evaluation at the clonal stage during the 2019–2020 season. Overall, 272 accessions succumbed to disease pressure during the evaluation period: 92 accessions at 3 months, 6 accessions at 6 months, and 174 accessions at 12 months (Figure 2). Hence, 297 accessions survived until root necrosis assessment at 12MAP. CBSD severity scores ranged from 1 to 4 at 3MAP and 6MAP and 1 to 5 at 12MAP (Figure 5). The percentage CBSD incidence ranged from 0 to 100 at 3MAP, 6MAP, and 12MAP. Mean scores for CBSD severity at 3MAP, 6MAP, and 12MAP were 2.3, 2.5, and 2.5, respectively. Mean scores of 79.7 and 84.4 were also recorded for CBSD foliar incidence and 59.3 for root incidence (Table 1). Approximately 80% of the genotypes had CBSD scores between 2 and 3 (Figure 5). In contrast, approximately 30% of the accessions had CBSD root necrosis scores ranging from 1 to 2. For CBSD incidence, more than 90% of the genotypes exhibited incidence scores between 75 and 100% at 3 and 6 months, while 25% of the genotypes registered root incidences between 75 and 100% for CBSD_*ri*_. For CMD, more than 90% of the genotypes exhibited severity scores between 1 and 2, and incidences between 0 and 25% at 6MAP (Figure 6).

**TABLE 1:** Field response of elite Nigerian clones in Uganda.

Mean squares												

SOV	Df	Severity					Incidence					HI
		CBSD_3*s*_	CBSD_6*s*_	CBSD_*rs*_	CMD_3*s*_	CMD_6*s*_	CBSD_3*i*_	CBSD_6*i*_	CBSD_*ri*_	CMD_3*i*_	CMD_6*i*_	
Genotype	292	0.209***	0.197**	1.371***	0.263***	0.408***	296***	218ns	1181***	748.1***	881.1***	0.027**
Check	2	21.440***	22.206***	68.053***	0.046***	0.012*	62369***	50893***	43000***	320.9**	95.6**	0.196***
Check vs. Genotype	1	44.463***	42.187***	1.240*	1.611***	3.109***	167756***	140320***	10866***	4243.1***	6803.1***	1.145***
Block	23	0.057ns	0.105ns	0.225ns	0.005ns	0.005ns	149ns	370ns	317ns	41.4ns	28.5	0.012ns
Residual	46	0.05	0.096	0.193	0.006	0.003	118	340	237	45.5	18.3	0.123
mean		2.306	2.478	2.498	1.383	1.499	79.76	84.41	59.32	20.35	22.42	0.28
CV (%)		9.4	12.2	17.5	6.5	4.8	13.1	21.4	25.8	72.7	42.2	40.5
H^2^		0.69	0.6	0.71	0.76	0.81	0.68	0.5	0.66	0.79	0.81	0.55

SOV, source of variation; Df, degrees of freedom; CBSD_3s_, CBSD severity at 3 months after planting (MAP); CBSD_6s_, CBSD severity at 6MAP; CBSD_3i_, CBSD incidence at 3MAP; CBSD_6i_, CBSD incidence at 6MAP; CMD_3s_, CMD severity at 3MAP; CMD_6s_, CMD severity at 6MAP; CMD_3i_, CMD incidence at 3MAP; CMD_6i_, CMD incidence at 6MAP; CBSD_rs_, CBSD root severity; CBSD_ri_, CBSD root incidence; HI, harvest index; CV, coefficient of variation; H^2^, entry mean broad-sense heritability; ns, non-significant at alpha = 0.05; *p ≤ 0.05; **p ≤ 0.01; ***p ≤ 0.00.

### Correlations Between Cassava Brown Streak Disease and Other Related Traits in the Seedling Trial

Foliar severity at six months had a significant but low positive correlation with CBSD root severity (*r* = 0.1376^**^) and CBSD root incidence (0.1223^**^) (Table 2). However, CBSD root severity positively correlated with CBSD root incidence (*r* = 0.8296^***^). There were no correlations between CBSD and CMD. However, CMD severity at six months had a low negative correlation with root weight (*r* = −0.1306^**^). Furthermore, root weight was negatively and marginally correlated with CBSD root necrosis incidence and root necrosis severity (−0.0946* and −0.1782^***^, respectively). There were marginal correlations between total carotenoid content and root weight (0.1894^***^). There were also marginal negative correlations between total carotenoids and CBSD root severity (−0.0612).

**TABLE 2:** Relationship between CBSD and other related traits in Nigerian genotypes evaluated in seedling trial at Namulonge in 2018.

Variables	CBSD_*ri*_	CBSD_*rs*_	CBSD_3*s*_	CBSD_6*s*_	CMD_3*s*_	CMD_6*s*_	Fresh_*RW*_	ttl_caro
CBSD_*ri*_	–							
CBSD_*rs*_	0.8296***	–						
CBSD_3*s*_	−0.0365ns	−0.019ns	–					
CBSD_6*s*_	0.1223**	0.1376**	−0.0222ns	–				
CMD_3*s*_	0.0766ns	0.0594ns	0.0199ns	0.0214ns	–			
CMD_6*s*_	0.0277ns	0.0718ns	−0.0316ns	−0.0331ns	0.0334ns	–		
Fresh_*RW*_	−0.0946*	−0.1782***	−0.0119ns	0.0076ns	0.0066ns	−0.1306**	–	
Total_Caro	0.0294ns	−0.0612ns	−0.0465ns	0.0217ns	0.0065ns	0.0588ns	0.1894***	–

***Significant (p < 0.001); **significant (p < 0.01); *significant (p < 0.05); CBSD_3s_, CBSD foliar severity at 3MAP; CBSD_6s_, CBSD foliar severity at 6MAP; CBSD_ri_, CBSD root incidence at 12MAP; CBSD_rs_, CBSD root severity at 12MAP; CMD_3s_, CMD severity at 3MAP; CMD_6s_, CMD severity at 6MAP; Fresh_RW_, fresh root weight; ttl_caro, total carotenoid content; ns, Not significant.

### Correlations Between Cassava Brown Streak Disease and Other Related Traits at the Clonal Evaluation

Correlations between CBSD and other agronomic traits evaluated at the clonal stage are presented in Table 3. Analyses between traits showed a significant positive relationship between CBSD root incidence and CBSD root severity (*r* = 0.90^***^). CBSD foliar severity at 3 months and CBSD foliar incidence at three months (CBSD_3*i*_) also had highly significant positive correlations of *r* = 0.89^***^. The same pattern was noted for CBSD foliar incidence at six months and CBSD foliar severity at six months (*r* = 0.89^***^). CBSD foliar incidence at three months also correlated positively and significantly with CBSD foliar incidence at six months (*r* = 0.86^***^) and CBSD foliar severity at six months (*r* = 0.78^***^). However, correlations were lower between CBSD root incidence (*r* = 0.40^***^) and CBSD root severity (*r* = 0.34^***^). Similarly, CBSD foliar severity at 3 months correlated positively with CBSD foliar incidence at six months (*r* = 0.77^***^) and CBSD foliar severity at six months (*r* = 0.78^***^), but lower with CBSD root incidence (*r* = 0.40^***^) and CBSD root severity (*r* = 0.35^***^). In contrast, CBSD foliar severity at three months correlated negatively with CMD incidence at three months (CMD_3*i*_) (*r* = −0.25^***^), CMD severity at 3 months (*r* = −0.25^***^), CMD incidence at six months (CMD_6*i*_) (*r* = −0.20^***^), and CMD severity at six months (*r* = −0.21^***^).

**TABLE 3:** Relationship between CBSD and other related traits in Nigerian genotypes evaluated in the clonal trial at Namulonge in 2019.

Variables	CBSD_*ri*_	CBSD_*rs*_	CBSD_3*i*_	CBSD_3*s*_	CBSD_6*i*_	CBSD_6*s*_	CMD_3*i*_	CMD_3*s*_	CMD_6*i*_	CMD_6*s*_	Fresh_*RW*_	HI	ttl_caro
CBSD_*ri*_	–												
CBSD_*rs*_	0.897***	–											
CBSD_3*i*_	0.4007***	0.3406***	–										
CBSD_3*s*_	0.3989***	0.3449***	0.8872***	–									
CBSD_6*i*_	0.3775***	0.3322***	0.8567***	0.7702***	–								
CBSD_6*s*_	0.4175***	0.3851***	0.7797***	0.7768***	0.8912***	–							
CMD_3*i*_	−0.1287ns	−0.0836ns	−0.1551ns	−0.2539***	−0.0087ns	−0.119ns	–						
CMD_3*s*_	−0.1279ns	−0.0875ns	−0.1578ns	−0.2501***	−0.0161ns	−0.116ns	0.9747***	–					
CMD_6*i*_	−0.1185ns	−0.0977ns	−0.1055ns	−0.2001***	0.0211ns	−0.0859ns	0.9257***	0.9076***	–				
CMD_6*s*_	−0.1127ns	−0.0899ns	−0.1181ns	−0.2081***	0.013ns	−0.0907ns	0.9073***	0.9235***	0.9695***	–			
Fresh_*RW*_	−0.2084***	−0.2189***	−0.5432***	−0.4915***	−0.5182***	−0.452***	−0.1031ns	−0.1185ns	−0.1218ns	−0.1355ns	–		
HI	−0.1699ns	−0.1672ns	−0.3658***	−0.352***	−0.3764***	−0.3367***	−0.0695ns	−0.0749ns	−0.0995ns	−0.1112ns	0.5092***	–	
ttl_caro	0.0337ns	0.0118ns	0.2067***	0.1533ns	0.17ns	0.1194ns	0.1006ns	0.1092ns	0.1077ns	0.1139ns	−0.078ns	−0.0015ns	–

***Significant (p < 0.001); CBSD_3s_, CBSD severity at 3MAP; CBSD_6s_, CBSD severity at 6MAP; CBSD_3i_, CBSD incidence at 3MAP; CBSD_6i_, CBSD incidence at 6MAP; CMD_3s_, CMD severity at 3MAP; CMD_6s_, CMD severity at 6MAP; CMD_3i_, CMD incidence at 3MAP; CMD_6i_, CMD incidence at 6MAP; CBSD_rs_, CBSD root severity; CBSD_ri_, CBSD root incidence; HI, harvest index; Fresh_RW_, fresh root weight during harvest; ttl_caro, total carotenoid content; ns, Not significant.

Fresh root weight during harvest had a significant negative correlation with CBSD foliar incidence at 3 months (*r* = −0.54^***^), CBSD foliar severity at 3 months (*r* = −0.49^***^), CBSD foliar incidence at 6 months (*r* = −0.52^***^), CBSD foliar severity at 6 months (*r* = −0.45^***^), CBSD root incidence (*r* = −0.21^***^), and CBSD root severity (*r* = −0.22^***^). However, there was a significant positive correlation between FRW and harvest index (*r* = 0.51^***^). There was a slight non-significant negative correlation between harvest index and CBSD root incidence (*r* = −0.17). CBSD root severity also had a slight negative non-significant correlation with harvest index (*r* = −0.17). Negative correlations were consistently observed between harvest index and CBSD foliar incidence at 3 months (*r* = −0.37^***^), CBSD foliar severity at 3 months (*r* = −0.36), CBSD foliar incidence at six months (*r* = −0.38), and CBSD foliar severity at six months (*r* = −0.34).

### Correlations Between Cassava Brown Streak Disease and Cassava Mosaic Disease at Both Seedling Evaluation Trial and Clonal Evaluation Trial

Correlations between seedling and clonal evaluations are presented in Table 4. There were significant correlations for CBSD_*ri*_ at both SET and CET (*r* = 0.48^***^) (Table 4). Correlations between CBSD_*rs*_ at SET and CET were also significant (*r* = 0.53^***^). CMD_6*s*_ score at the seedling stage was positively correlated with CMD_3*s*_ at the clonal stage (*r* = 0.68^***^) and CMD_6*s*_ at the clonal stage (*r* = 0.67^***^). Negative correlations were consistently observed between CMD_6*s*_ at the seedling stage and CBSD_3*s*_ at the clonal stage (*r* = −0.36^***^), and also with CMD_6*s*_ at the seedling stage and CBSD_6*s*_ at the clonal stage (*r* = 0.28^***^). There were slightly non-significant negative correlations between S_CMD_3*s*_ and C_CBSD_3*s*_ (*r* = −0.07), C_CBSD_6*s*_ (*r* = −0.05), and C_CBSD_*rs*_ (*r* = −0.03) (Table 4). There were also slightly positive non-significant correlations between S_CBSD_3*s*_ and C_CBSD_3*s*_ (*r* = 0.05), S_CBSD_3*s*_ and C_CBSD_*rs*_ (*r* = 0.01), and S_CBSD_3*s*_ and C_CBSD_*ri*_ (*r* = 0.03).

**TABLE 4:** Relationships between CBSD and CMD for the genotypes between the seedling and clonal trial.

Variables	C_CBSD_3*s*_	C_CBSD_6*s*_	C_CBSD_*ri*_	C_CBSD_*rs*_	C_CMD_3*s*_	C_CMD_6*s*_	S_CBSD_3*s*_	S_CBSD_6*s*_	S_CBSD_*ri*_	S_CBSD_*rs*_	S_CMD_3*s*_	S_CMD_6*s*_
C_CBSD_3*s*_	–											
C_CBSD_6*s*_	0.6443***	–										
C_CBSD_*ri*_	0.2165	0.1245	–									
C_CBSD_*rs*_	0.1346	0.0837	0.8921***	–								
C_CMD_3*s*_	−0.4973***	−0.3536***	–0.1537	–0.1586	–							
C_CMD_6*s*_	−0.4606***	−0.335***	–0.0785	–0.1149	0.922***	–						
S_CBSD_3*s*_	0.0472	0.0051	0.0364	–0.0182	–0.0037	–0.021	–					
S_CBSD_6*s*_	0.0538	0.1266	–0.1364	–0.1313	0.0963	0.1498	0.0057	–				
S_CBSD_*ri*_	0.0881	0.0839	0.475***	0.5067***	–0.1733	–0.1527	–0.1021	0.156	–			
S_CBSD_*rs*_	0.0838	0.0426	0.4326***	0.5266***	–0.0863	–0.0609	–0.0977	0.1978	0.8591***	–		
S_CMD_3*s*_	–0.0659	–0.0536	0.0552	–0.0332	0.2315	0.3353***	0.152	0.0921	–0.0491	–0.0529	–	
S_CMD_6*s*_	−0.3556***	−0.2803***	–0.0661	–0.0882	0.6781***	0.6653***	–0.0638	0.0705	0.0185	0.0969	0.1991	–

***Significant (p < 0.001); S_CBSD_3s_, Seedling evaluation for CBSD severity at 3MAP; S_CBSD_6s_, Seedling evaluation for CBSD severity at 6MAP; S_CMD_3s_, Seedling evaluation for CMD severity at 3MAP; S_CMD_6s_, Seedling evaluation for CMD severity at 6MAP; S_CBSD_rs_, Seedling evaluation for CBSD root severity at 12 months; S_CBSD_ri_, Seedling evaluation for CBSD root incidence at 12 months. C_CBSD_3s_, Clonal evaluation for CBSD severity at 3MAP; C_CBSD_6s_, Clonal evaluation for CBSD severity at 6MAP; C_CMD_3s_, Clonal evaluation for CMD severity at 3MAP; C_CMD_6s_, Clonal evaluation for CMD severity at 6MAP; C_CBSD_rs_, Clonal evaluation for CBSD root severity at 12 months; C_CBSD_ri_, Clonal evaluation for CBSD root incidence at 12 months. Correlations were obtained using 294 genotypes with complete dataset for CBSD and CMD at SET and CET.

### Variation, Heritabilities for Cassava Brown Streak Disease and Other Important Traits at the Clonal Evaluation

Analysis of variance (ANOVA) results showed significant differences among test genotypes for CBSD severity at 3 (*p* ≤ 0.001), 6 (*p* ≤ 0.01), and 12 months (*p* ≤ 0.001) (Table 1). There were also significant differences among test genotypes for CBSD incidence at 3 and 12 months (*p* ≤ 0.001) but not at 6 months. Comparisons between test genotype means and means of the checks also showed significant differences (*p* ≤ 0.05 and *p* ≤ 0.001) for both CBSD incidence and severity, except CBSD_*rs*_ (*p* ≤ 0.05), which exhibited significant marginal differences.

Entry mean-based broad-sense heritability estimates ranged from moderate to high (Table 1). Overall, disease traits had higher heritability estimates than agronomic traits. For disease traits, CBSD_6*i*_ and CBSD_6*s*_ had the lowest heritability estimates with moderate *H*^2^ of 0.50 and 0.60, respectively. CMD severity and incidence at 6 months had the highest estimates of heritability with *H^2^* = 0.81 and 0.81, respectively. Heritability estimates for CBSD (incidence and severity) at 3, 6, and 12 months varied from moderate to high (CBSD_3*i*_ *H*^2^ = 0.68, CBSD_6*i*_ *H^2^* = 0.50, CBSD_*ri*_ *H^2^* = 0.66, and CBSD_3*s*_ *H^2^* = 0.79, CBSD_6*s*_ *H*^2^ = 0.60, CBSD_*rs*_ *H^2^* = 0.71).

### Comparisons and Ranking of Cassava Clones Based on the Selection Index

Genotype ranking based on the non-weighted rank summation SI revealed the overall best performer as UGIN181367, a yellow-fleshed clone (Table 5). Other top nine performing genotypes included UGIN180528, UGIN181535, UGIN180247, UGIN181791, UGIN181326, UGIN181154, UGIN181371, UGIN180602, and UGIN181346. Notably, 8 out of the best 15 clones were yellow clones. Families of the best and worst performers in terms of CBSD resistance are presented in Table 6. Only family F036 had 2 progenies in the best 15 categories, and other families, such as F001, F004, F014, and F018, had one progeny each in the best performing category.

**TABLE 5:** Rankings of the best-15 and worst-15 genotypes following CBSD evaluations at Namulonge in 2019.

Best-15 performing genotypes									Worst-15 performing genotypes								
Genotype	CBSD_3*s*_	CBSD_6*s*_	CBSD_*rs*_	CBSD_3*i*_	CBSD_6*i*_	CBSD_*ri*_	SI	ttl_caro	Genotype	CBSD_3*s*_	CBSD_6*s*_	CBSD_*rs*_	CBSD_3*i*_	CBSD_6*i*_	CBSD_*ri*_	SI	ttl_caro
UG110017	1	1	1	1	1	1	6	1	UGN180552	278	296	281	296	276	224	1651	2
Mkumba	2	2	9	2	2	18	35	1	UGIN180141	294	257	284	260	261	275	1631	1
UGIN181367	8	12	32	9	13	28	102	2	UGIN181204	295	258	283	259	260	274	1629	1
UGIN180528	9	6	26	27	5	33	106	1	UGIN180263	273	282	245	289	296	207	1592	1
UGIN181535	12	61	13	20	26	10	142	1	UGIN181764	277	252	268	295	275	223	1590	1
UGIN180247	6	5	46	10	7	71	145	4	UGIN181352	289	256	248	257	258	272	1580	1
UGIN181791	5	107	3	8	62	2	187	1	UGIN181162	284	269	293	195	239	280	1560	3
UGIN181326	4	38	6	4	141	4	197	1	UGIN181784	288	270	278	194	238	279	1547	1
UGIN181154	18	11	66	52	9	50	206	2	UGIN180201	282	268	260	192	237	278	1517	1
UGIN181371	15	14	94	16	16	63	218	3	UGIN181592	292	165	266	258	259	273	1513	3
UGIN180602	17	57	4	35	102	8	223	4	UGIN180481	285	234	272	248	173	286	1498	2
UGIN181346	11	148	8	12	51	9	239	1	UGIN181454	291	235	265	247	172	285	1495	1
UGIN180416	25	45	33	105	18	29	255	1	UGIN181343	283	255	161	255	256	271	1481	1
UGIN181466	14	46	11	17	186	6	280	3	UGIN181598	221	281	218	288	295	178	1481	2
UGIN181723	23	99	61	30	37	40	290	2	UGIN181503	266	289	285	133	267	227	1467	1
UGIN181678	19	94	14	41	121	11	300	1									
UGIN180342	26	19	82	26	87	60	300	3									

*CBSD_3s_, CBSD severity at 3MAP; CBSD_3i_, CBSD incidence at 3MAP; CBSD_6s_, CBSD severity at 6MAP; CBSD_6i_, CBSD incidence at 6MAP CBSD_ri_, CBSD root incidence at 12MAP; CBSD_rs_, CBSD root severity at 12MAP; SI, rank summation index; ttl_caro, total carotenoid content; Mkumba, CBSD resistant/tolerant check; UG110017, resistant/tolerant check; TME 204, CBSD susceptible check. Checks were included to enable direct comparisons with test genotypes. The non-weighted rank-summation SI was used in ranking.*

**TABLE 6:** Family based responses to CBSD of the best-15 and worst-15 Nigerian genotypes evaluated at Namulonge in 2019.

Families of the Best-15 genotypes					Families of the Worst-15 genotypes				
Clones	Family	Progenitors	SI	ttl_caro	Clones	Family	Progenitors	SI	ttl_caro
UGIN180416	F001	F153P046 × F58P008	255	1	UGIN180141	F005	F50P003 × F58P008	1631	1
UGIN180342	F004	F153P046 × F58P008	300	3	UGIN180481	F008	F153P046 × F154P012	1498	2
UGIN180247	F014	F168P002 × F63P006	145	4	UGIN180201	F010	F154P011 × F10P008	1517	1
UGIN180528	F018	F153P053 × F58P008	106	1	UGIN180263	F015	F50P003 × F58P002	1592	1
UGIN180602	F022	F154P012 × F163P008	223	4	UGIN180552	F020	F153P014 × F63P014	1651	2
UGIN181367	F036	F152P004 × F63P006	102	2	UGIN181162	F051	F12P061 × F162P008	1560	3
UGIN181371	F036	F152P004 × F63P006	218	3	UGIN181204	F054	F77P003 × F58P008	1629	1
UGIN181154	F051	F12P061 × F162P008	206	2	UGIN181352	F064	F158P046 × F11P011	1580	1
UGIN181326	F063	F58P008 × F122P006	197	1	UGIN181343	F064	F158P046 × F11P011	1481	1
UGIN181346	F064	F158P046 × F11P011	239	1	UGIN181454	F069	F165P009 × F2P030	1495	1
UGIN181466	F070	F87P016 × F64P001	280	3	UGIN181503	F072	F58P008 × F91P001	1467	1
UGIN181535	F075	F26P004 × F10P007	142	1	UGIN181592	F079	F153P046 × F58P013	1513	3
UGIN181678	F084	F91P011 × F162P008	300	1	UGIN181598	F079	F153P046 × F58P013	1481	2
UGIN181723	F088	F124P001 × F63P014	290	2	UGIN181764	F090	F153P046 × F10P008	1590	1
UGIN181791	F093	F124P001 × F162P008	187	1	UGIN181784	F092	F165P009 × F124P001	1547	1

*SI, Selection index; ttl_caro, total carotenoid content. Out of 106 families, a total of 24 had progenies among the best 30 performers. The non-weighted rank-summation SI was used in ranking.*

**FIGURE 7:**
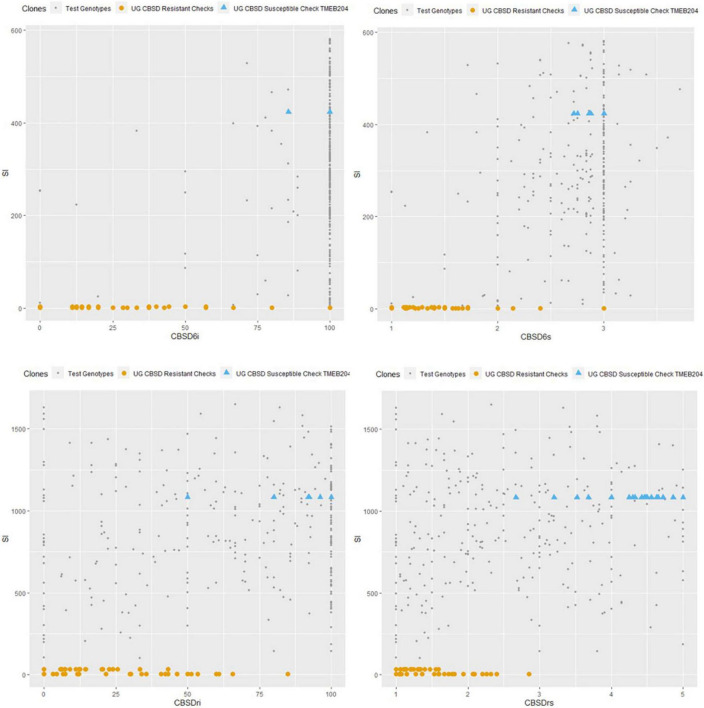
Comparison of CBSD susceptible and resistant checks to test genotypes at 6 and 12 months. CBSD_6*i*_, CBSD incidence at 6MAP; CBSD_6*s*_, CBSD severity at 6MAP; CBSD_*ri*_, CBSD root incidence; CBSD_*rs*_, CBSD root severity; SI, Selection Index.

Comparison of checks and test genotypes is shown in Figure 7. CBSD-resistant checks (Mkumba and NAROCASS 1) performed markedly better than all other test genotypes for CBSD resistance. However, 65% of test genotypes performed better than the susceptible check (TMEB204) in terms of CBSD resistance (Figure 7).

From the seedling to clonal evaluation stage, family progression with regard to CBSD is presented in Supplementary Figure 1. Mean CBSD severities at 3, 6, and 12 months in the seedling stage were 1.03, 1.18, and 2.28, respectively. However, at the clonal stage, mean CBSD severities at 3, 6, and 12 months were 2.50, 2.70, and 2.57, respectively. Evidently, there was a general increase in CBSD from seedling to clonal stage. The survival rate of progenies per family from seedling to clonal evaluation ranged from 5 to 80% (Supplementary Figure 2). The family F059 had the highest survival rate, with 80% of its progenies surviving until harvest at 12MAP. Only two families had higher CBSD root severity means than that of the susceptible check TME 204 (4.33) at 12 months, with F040 being the most susceptible with a mean score of 5.0. Progenies per family for the seedling trial ranged from 10 to 130. In contrast, progenies advanced to CET after selection ranged from 1 to 18, averaging four progenies per family. Overall, 30 families succumbed to CBSD pressure during the seedling evaluation.

## Discussion

Cassava brown streak disease is one of the topmost threats to food security (Patil et al., 2015), undermining any investments made to maximize the benefits of cassava value chains. For this reason, concerted efforts are devoted toward finding sustainable disease management options. Based on the lessons learned in East Africa, where less attention was given to CBSD until it reached epidemic levels after several decades of existence at low altitudes of coastal areas in Tanzania (Storey, 1936; Legg et al., 2011, 2015), it has become necessary to take preemptive measures to prevent the spread of CBSD in West Africa, a major cassava-producing zone in Africa, and limit its impact. Accordingly, this study aimed at contributing toward development of improved cassava varieties with enhanced CBSD resistance in Nigeria.

Consequently, 1,980 seedlings were evaluated for their responses to CBSD. Of these, 569 clones were selected for further evaluation at the clonal level. By the first year of field evaluation, 38% of the 1,980 seedlings succumbed to CBSD pressure during the first three months after planting, and by the 12th month, only 46% of the genotypes survived for root necrosis assessment. This magnitude of CBSD severity can be attributed to a lack of CBSD resistance alleles in the Nigerian germplasm. A similar outcome was noted by Elegba et al. (2020), who reported less than 50% survival rate for Ghanaian cassava genotypes tested against CBSV and UCBSV under screen-house conditions. Likewise, Winter et al. (2019) reported moderate to severe foliar symptoms when 238 South American cassava genotypes were assessed for resistance to CBSVs. Viral inoculum buildup occurs when diseased stem cuttings are recycled (Kaweesi et al., 2014). Indeed, significant correlations between seedling and clonal CBSD root incidences and severities (S_CBSD_*ri*_ and C_CBSD_*ri*_ *r* = 0.48^***^; S_CBSD_*rs*_ and C_CBSD_*rs*_ *r* = 0.53^***^) suggest viral inoculum buildup in the course of the evaluations. For these reasons, conclusions cannot be deducted on resistance status at seedling trials.

Progenies per family ranged from 10 to 130 in the SET, with an average of 18 progenies per family. In CET, this varied between 1 and 18, with an average of four progenies per family (Supplementary Figure 1). This variation in the number of progenies from each progenitor is related to the highly erratic flowering behavior of different cassava genotypes (Ceballos et al., 2012). Overall, family averages at 6 and 12 months in the CET indicated that most families had considerably low resistance to CBSD owing to high susceptibility scores (Supplementary Figure 1). Some families had seedlings that exhibited marginal CBSD symptoms. A good example is family F036, which had two of its progenies (UGIN181367 and UGIN181371) among the best performers for CBSD at CET. This situation presents an opportunity to select superior genotypes for further evaluation of traits of interests. Similar reports suggested by Mrema (2016) also highlighted on cassava families with varying number of seedlings that exhibited CBSD symptoms and seedlings that were symptomless.

A similar phenomenon was reported by Shirima et al. (2019), who noted the degeneration of cassava genotypes due to increased viral inoculum resulting from stem recycling. Escape variants underscore why the selection for CBSD resistance among previously untested genotypes should not focus on seedling evaluations alone. This is because, at SET, the viral load in stem cuttings would be low compared to that in the clonal level. Also, seedling evaluations and data collection are based on a single plant, as opposed to 10 plants at the clonal stage. Thus, the evaluation at the clonal stage allows for better phenotypic data computation and exposes escape variants, such as the case of UGIN180447 and other accessions that exhibited marginal CBSD symptoms in the seedling trial.

Significant differences were recorded among test genotypes for all traits in the CET, except CBSD incidence at six months (Table 1). A mean score of 84.4 for CBSD_6*i*_ indicates that most genotypes exhibited high levels of CBSD at six months, explaining why there were no significant variations among the test genotypes. This reinforces the lack of resistance alleles in the Nigerian germplasm. Significant differences in CBSD root severity at three, six, and 12 months, however, indicate differential responses of the genotypes. Similar findings were reported by Abaca et al. (2012), Kaweesi et al. (2014), and Pariyo et al. (2015), who identified varied resistance/susceptibility categorizations of foliar and root severities. This observation is in accordance with earlier studies by Jennings (1960) and Hillocks and Thresh (2000), who reported that different cultivars respond differently to CBSD.

For the clonal trial, the significant positive correlations between CBSD shoot severity and incidence, with CBSD root severity and incidence (Table 3), agree with the reports suggested by Abaca et al. (2012) and Mrema (2016). Results also indicated negative correlations between CBSD and CMD, which were largely non-significant except for CBSD_3*s*_ (Table 3). The negative correlation suggests that the traits were under the control of different genetics. The negative correlations between harvest index and CBSD illustrate the damage inflicted by CBSD, as observed in previous studies by Nuwamanya et al. (2015) and Mrema (2016). The non-significant negative correlation between harvest index and CMD is likely due to high CMD resistance among the evaluated germplasm. CMD resistance breeding has been ongoing in Nigeria since 1932 (Hahn et al., 1980). Thus, most of the Nigerian germplasm was resistant as evidenced by mean severities and incidences of about 1.4 and 20% respectively (Table 1).

A non-weighted rank summation SI, which comprised the summation of genotype rank for CBSD incidence and severity data at three, six, and 12 months, was used to rank the performance of the test genotypes (Table 5). UGIN181367 (SI = 102) was the overall best genotype in its response to CBSD. This genotype exhibited marginal symptoms to CBSD foliar and root severity and incidence at both SET and CET. However, UGIN181367 was ranked third after NAROCASS 1 and Mkumba, the two resistant checks. NAROCASS 1 and Mkumba had SI values of 6 and 35, respectively. They are varieties that exhibit high tolerance to CBSD (Mukiibi et al., 2019). Interestingly, a few other genotypes, including UGIN180528, UGIN181535, UGIN180247, also had a high SI ranking and exhibited marginal symptoms for CBSD foliar and root severity and incidence (Table 5). These identified genotypes merit for further evaluations to confirm their CBSD responses.

Broad-sense heritability estimates ranged from moderate to high for all traits in the clonal trial (Table 1). Except for CBSD_*rs*_ and CBSD_*ri*_, heritability estimates of CBSD incidence and severity at six and 12 months were comparable to those presented by Kayondo et al. (2018) and Ozimati et al. (2018) (CBSD_*rs*_ *H^2^* = 0.25 and 0.26, respectively). The heritability estimates for CBSD_*rs*_ in the clonal trial were also similar to the reports suggested by Okul Valentor et al. (2018) (*H^2^* = 0.69). Estimates of broad-sense heritability for CBSD incidence were generally lower than those of CBSD severity in the clonal trial (Table 1). This contrasts with the findings suggested by Okul Valentor et al. (2018), who reported higher broad-sense heritability estimates for CBSD incidence. Accordingly, heritability estimates for CBSD_*s*_ and CBSD_*i*_ in the clonal trials suggest that effective selection can be made at the clonal stage.

## Conclusion

This study was initiated on the premise of determining the field reaction of Nigerian cassava genotypes to CBSD. Therefore, this could inform the extent of CBSD resistance/susceptible allele distribution in the elite Nigerian germplasm used for cultivation and/or genetic improvement. Based on the generated datasets, it can be concluded that most of the genotypes that constituted the Nigerian cassava population exhibited significant susceptibility to CBSD within the 2-year evaluation period. Fortunately, 15 clones have been identified to have either limited or no CBSD symptoms. These genotypes can be re-evaluated at higher plot capacities and in diverse sites to confirm their resistance/tolerance to CBSD. In addition, crossing the 15 identified clones with CBSD-resistant Ugandan cassava varieties would ensure the introduction of more resistance alleles into the genome of the Nigerian population. The introgression of CBSD resistance from East African clones is, therefore, critical as a preemptive breeding strategy for CBSD resistance in Nigeria. This will help the Nigerian cassava breeding program to develop varieties with more durable resistance to the disease. Furthermore, continued screening and characterization of Nigerian cassava germplasm for responses to CBSD infection is important to identify more genotypes with CBSD tolerance or resistance traits. Deploying such tolerant or resistant cultivars can help in the effective control of the disease spread and reduction in losses associated with it.

## Data Availability Statement

The original contributions presented in the study are included in the article/Supplementary Material, further inquiries can be directed to the corresponding author.

## Author Contributions

CA was involved in conceptualization, data collection, data analysis, and manuscript writing. MO-S was involved in conceptualization, manuscript writing, manuscript editing, and supervision. AI and DN were involved in manuscript editing and supervision. AO involved in the data collection, data analysis, and manuscript editing. PG was involved in conceptualization, supervision, and manuscript editing. RK was involved in conceptualization, data collection, data analysis, supervision, manuscript writing, and manuscript editing. JO was involved in conceptualization, manuscript editing, fund acquisition, and supervision. CE was involved in conceptualization, manuscript writing, manuscript editing, fund acquisition, and supervision. All authors contributed to the article and approved the submitted version.

## Conflict of Interest

The authors declare that the research was conducted in the absence of any commercial or financial relationships that could be construed as a potential conflict of interest.

## Publisher’s Note

All claims expressed in this article are solely those of the authors and do not necessarily represent those of their affiliated organizations, or those of the publisher, the editors and the reviewers. Any product that may be evaluated in this article, or claim that may be made by its manufacturer, is not guaranteed or endorsed by the publisher.
