# Supplementary material for: Cassava Brown Streak Disease Response and Association With Agronomic Traits in Elite Nigerian Cassava Cultivars
Source: Front Plant Sci. 2021 Nov 22;12:720532. doi: 10.3389/fpls.2021.720532 (PMC8646096; doi:10.3389/fpls.2021.720532)
Supplement: Supplementary file 1 [file Data_Sheet_1.docx]

**Tables**

Supplementary Table 1. Pedigree of seedlings shipped and evaluated in Uganda.

| Pedigree of seedlings | | | | | | | |
| --- | --- | --- | --- | --- | --- | --- | --- |
|  | **Cross Combination** | |  |  | **Cross Combination** | |  |
| Family | **Mother** | **Father** | **No of Progeny** | **Family** | **Mother** | **Father** | **No of Progeny** |
| F001 | F153P046 | F58P008 | 130 | F036 | F152P004 | F63P006 | 40 |
| F002 | F153P014 | F63P006 | 40 | F037 | F63P005 | F154P012 | 10 |
| F003 | F58P008 | F63P006 | 40 | F038 | F91P011 | F153P046 | 10 |
| F004 | F153P046 | F62P001 | 30 | F039 | F91P011 | F152P004 | 20 |
| F005 | F50P003 | F58P008 | 30 | F040 | F27P004 | F124P001 | 10 |
| F006 | F153P046 | F58P002 | 30 | F041 | F163P005 | F2P030 | 10 |
| F007 | F163P005 | F162P008 | 40 | F042 | F62P008 | F2P030 | 20 |
| F008 | F153P046 | F154P012 | 30 | F043 | F58P002 | F58P008 | 10 |
| F009 | F153P046 | F63P014 | 90 | F044 | F163P005 | F63P014 | 10 |
| F010 | F154P011 | F10P008 | 20 | F045 | F50P0033 | F170P019 | 10 |
| F011 | F153P053 | F58P008 | 10 | F046 | F91P011 | F58P002 | 10 |
| F012 | F165P009 | F58P008 | 20 | F047 | F168P001 | F58P008 | 10 |
| F013 | F153P046 | F162P008 | 20 | F048 | F58P008 | F154P012 | 20 |
| F014 | F168P002 | F63P006 | 20 | F049 | F10P010 | F58P008 | 10 |
| F015 | F50P003 | F58P002 | 20 | F050 | F91P011 | F24P001 | 10 |
| F016 | F58P013 | F58P008 | 30 | F051 | F12P061 | F162P008 | 20 |
| F017 | F163P005 | F64P001 | 20 | F052 | F168P002 | F63P014 | 10 |
| F018 | F62P006 | F62P008 | 20 | F053 | F68P003 | F63P006 | 20 |
| F019 | F2P004 | F58P008 | 10 | F054 | F77P003 | F58P008 | 10 |
| F020 | F153P014 | F63P014 | 20 | F055 | F91P011 | F154P012 | 10 |
| F021 | F163P005 | F58P008 | 20 | F056 | F152P001 | F58P008 | 20 |
| F022 | F154P012 | F163P008 | 10 | F057 | F50P003 | F162P007 | 10 |
| F023 | F28P008 | F58P002 | 30 | F058 | F62P006 | F68P007 | 10 |
| F024 | F27P004 | F63P006 | 20 | F059 | F2P030 | F124P001 | 10 |
| F025 | F27P004 | F58P008 | 10 | F060 | F168P002 | F58P008 | 10 |
| F026 | F89P015 | F170P019 | 20 | F061 | F63P014 | F58P002 | 10 |
| F027 | F165P009 | F10P007 | 20 | F062 | F58P008 | F63P014 | 20 |
| F028 | F50P003 | F2P030 | 10 | F063 | F58P008 | F122P006 | 20 |
| F029 | F84P002 | F62P008 | 20 | F064 | F158P046 | F11P011 | 20 |
| F030 | F153P046 | F124P001 | 20 | F065 | F163P005 | F63P006 | 10 |
| F031 | F91P011 | F10P008 | 10 | F066 | F165P015 | F10P007 | 10 |
| F032 | F87P016 | F58P008 | 20 | F067 | F162P008 | F124P001 | 20 |
| F033 | F84P002 | F124P001 | 20 | F068 | F153P014 | F52P008 | 20 |
| F034 | F12P001 | F154P011 | 20 | F069 | F165P009 | F2P030 | 20 |
| F035 | F168P002 | F58P002 | 10 | F070 | F87P016 | F64P001 | 10 |

Supplementary Table 1. Pedigree of seedlings and evaluated in Uganda cont.

| Pedigree of seedlings | | | | | | | | |
| --- | --- | --- | --- | --- | --- | --- | --- | --- |
|  | **Cross Combination** | |  |  | **Cross Combination** | |  |  |
| Family | **Mother** | **Father** | **No of Progeny** | **Family** | **Mother** | **Father** | **No of Progeny** |  |
| F071 | F3P021 | F63P014 | 20 | F089 | F153P014 | F154P011 | 20 |  |
| F072 | F58P008 | F91P001 | 20 | F090 | F153P046 | F10P008 | 20 |  |
| F073 | F168P002 | F68P003 | 10 | F091 | F58P002 | F152P004 | 10 |  |
| F074 | F165P009 | F162P008 | 10 | F092 | F165P009 | F124P001 | 10 |  |
| F075 | F27P004 | F10P007 | 10 | F093 | F124P001 | F162P008 | 10 |  |
| F076 | F163P005 | F168P003 | 10 | F094 | F27P004 | F2P030 | 10 |  |
| F077 | F84P002 | F10P008 | 20 | F095 | F165P009 | F58P002 | 10 |  |
| F078 | F50P008 | F63P006 | 20 | F096 | F63P014 | F63P006 | 20 |  |
| F079 | F153P046 | F58P013 | 20 | F097 | F58P013 | F10P008 | 20 |  |
| F080 | F163P005 | F10P007 | 20 | F098 | F27P004 | F50P003 | 10 |  |
| F081 | F91P011 | F58P008 | 20 | F099 | F153P046 | F68P003 | 20 |  |
| F082 | F50P003 | F58P014 | 10 | F100 | F50P003 | F63P006 | 20 |  |
| F083 | F124P001 | F58P002 | 20 | F101 | F12P001 | F2P030 | 10 |  |
| F084 | F91P011 | F162P008 | 10 | F102 | F153P046 | F50P004 | 20 |  |
| F085 | F168P002 | F58P013 | 10 | F103 | F163P005 | F154P012 | 10 |  |
| F086 | F91P011 | F10P007 | 10 | F104 | F58P002 | F63P006 | 10 |  |
| F087 | F162P008 | F38P008 | 20 | F105 | F124P001 | F64P001 | 10 |  |
| F088 | F124P001 | F63P014 | 10 | F106 | F50P002 | F63P006 | 10 |  |

NB: Progenitors were selected owing to their per se performance for DMC, CMD resistance, and yield. These progenitors constitute part of Cycle one (C1) of the NRCRI elite germplasm

Supplementary Table 2. Summary table for seedling trial

| Statistics | Variables | | | | | | | |
| --- | --- | --- | --- | --- | --- | --- | --- | --- |
|  | **CBSD3s** | **CBSD6s** | **CBSDri** | **CBSDrs** | **CMD3s** | **CMD6s** | **FreshRW** | **ttl_caro** |
| Mini. Score | 1 | 1 | 0 | 1 | 1 | 1 | 0 | 1 |
| Median score | 1.0 | 1.0 | 62.5 | 2.0 | 1.0 | 1.0 | 0.8 | 2.0 |
| Mean Score | 1.03 | 1.21 | 56.8 | 2.30 | 1.12 | 1.44 | 1.24 | 2.09 |
| Maxi. Score | 3 | 5 | 100 | 5 | 5 | 4 | 8.6 | 6 |
| Std. Dev | 0.19 | 0.53 | 40.36 | 1.28 | 0.58 | 0.85 | 1.39 | 1.27 |
| CV | 18.89 | 44.15 | 71.05 | 55.54 | 51.91 | 59.15 | 111.64 | 60.48 |

CBSD3s = CBSD severity at 3MAP; CBSD6s = CBSD severity at 6MAP; CBSDri = CBSD root incidence; CBSDrs = CBSD root severity; CMD3s = CMD severity at 3MAP; CMD6s = CMD severity at 6MAP; FreshRW = Fresh root weight; ttl_caro = Total carotenoid content; Mini. Score = Minimum severity score; Maxi. Score = Maximum severity score; Std. Dev; Standard deviation; CV = Coefficient of variation

**Figures**


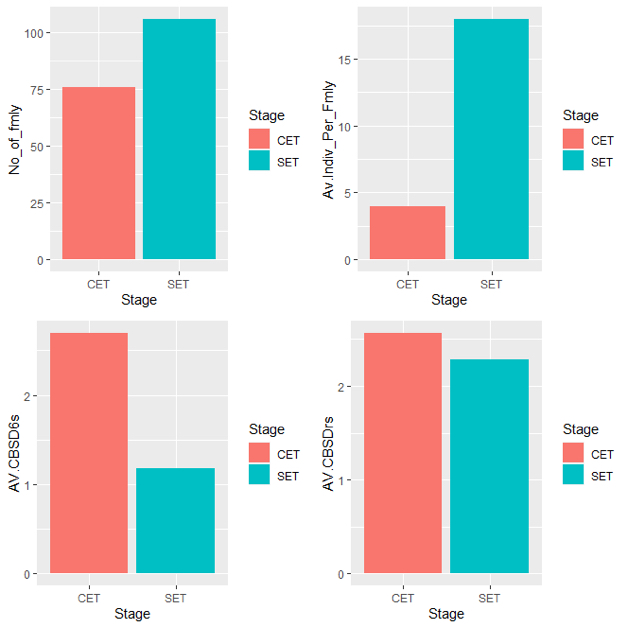


Supplementary Figure 1. Family progression from SET to CET. No_of_fmly = Number of represented families; Av.Indiv_Per_Fmly = Average number of individuals per family; AV.CBSD6s = Average cassava brown streak disease severity at six months; AV.CBSDrs = Average cassava brown streak disease root necrosis sevrity at twelve months.


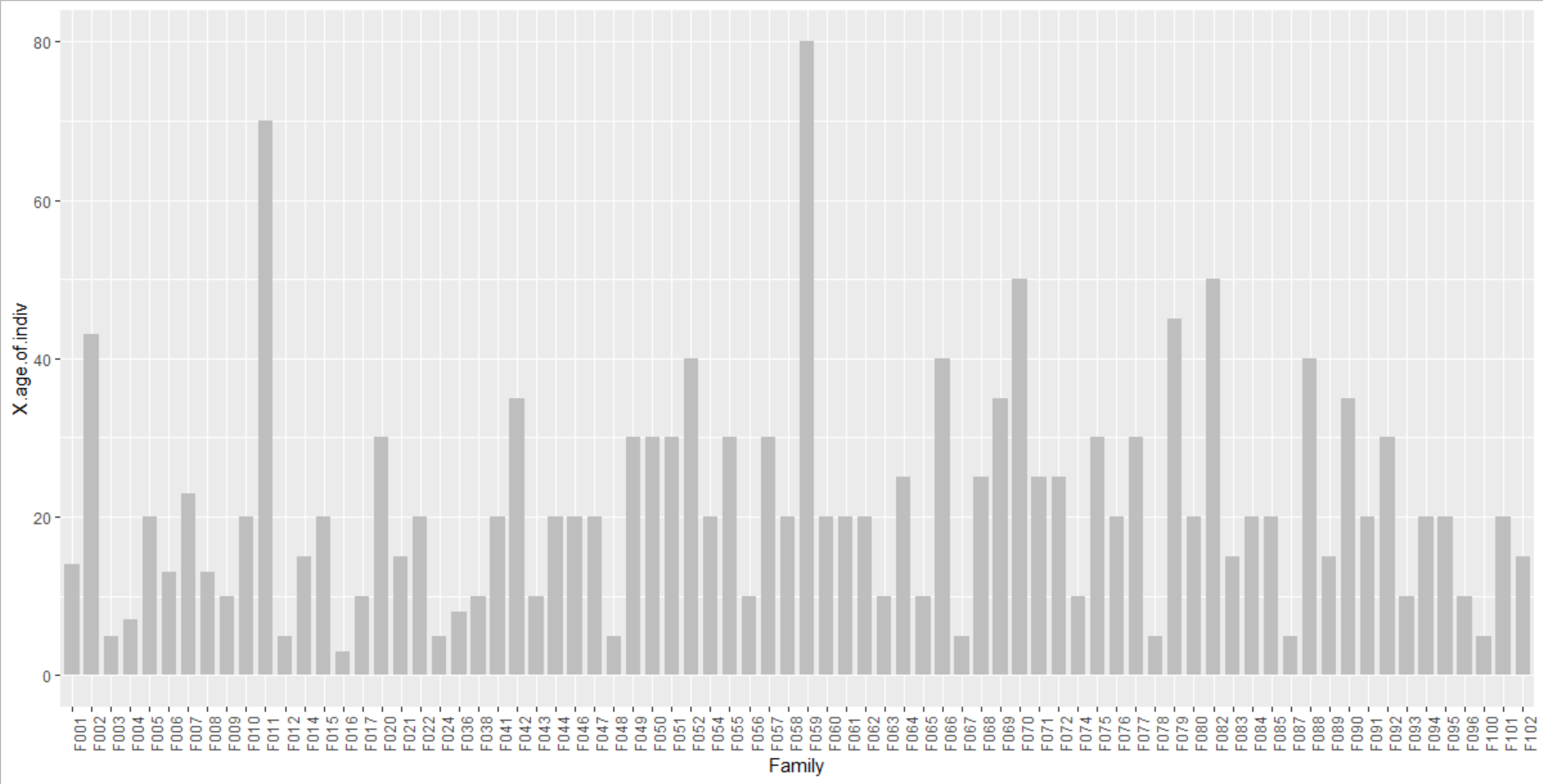


Survival rate of progenies (%)

Families

Supplementary Figure 2. Survival rate of progenies per family from SET to CET.
